# Increased peritoneal permeability at peritoneal dialysis initiation is a potential cardiovascular risk in patients using biocompatible peritoneal dialysis solution

**DOI:** 10.1186/1471-2369-15-173

**Published:** 2014-11-01

**Authors:** Yoshifumi Hamasaki, Kent Doi, Mototsugu Tanaka, Haruki Kume, Yoshitaka Ishibashi, Yutaka Enomoto, Toshiro Fujita, Yukio Homma, Masaomi Nangaku, Eisei Noiri

**Affiliations:** 22nd Century Medical and Research Center, The University of Tokyo Hospital, 7-3-1 Hongo, Bunkyo, Tokyo, Japan; Emergency and Critical Care Medicine, The University of Tokyo Hospital, Tokyo, Japan; Nephrology and Endocrinology, The University of Tokyo, 7-3-1 Hongo, Bunkyo, Tokyo, Japan; Urology, The University of Tokyo Hospital, Tokyo, Japan; Japanese Red Cross Medical Center, Tokyo, Japan; Mitsui Memorial Hospital, Tokyo, Japan; Division of Clinical Epigenetics, Research Center for Advanced Science and Technology (RCAST), The University of Tokyo, Tokyo, Japan

**Keywords:** Biocompatible peritoneal dialysis solution, Cardiovascular disease, Peritoneal dialysis, Peritoneal equilibration test, Peritoneal permeability

## Abstract

**Background:**

Cardiovascular disease is a frequent cause of death in peritoneal dialysis patients. Biocompatible peritoneal dialysis solutions with neutral pH have been anticipated to reduce cardiovascular disease more than conventional peritoneal dialysis solutions with low pH, but it remains unclear which factors at peritoneal dialysis initiation increase cardiovascular risk in patients using biocompatible peritoneal dialysis solutions. This study was undertaken to investigate which clinical factors at peritoneal dialysis initiation, including peritoneal transport status, are associated with cardiovascular event in patients using biocompatible peritoneal dialysis solution.

**Methods:**

This retrospective cohort study of peritoneal dialysis patients using biocompatible solutions with neutral pH assessed relations of clinical parameters at peritoneal dialysis initiation to cardiovascular event during the subsequent five years.

**Results:**

Of 102 patients who started peritoneal dialysis, cardiovascular event occurred in 18. Age, history of cardiovascular disease before peritoneal dialysis initiation, hemoglobin, serum albumin, C-reactive protein, peritoneal permeability defined by the ratio of dialysate to plasma creatinine concentration at 4 hr (D/Pcre) in peritoneal equilibration test (PET), number of patients in each PET category defined by D/Pcre, and peritoneal protein clearance significantly differed between patients with and without cardiovascular event. For patients divided according to PET category using Kaplan–Meier method, the group of high average to high peritoneal transporters exhibited significantly high incidence of cardiovascular event and mortality compared with the groups of low and low-average peritoneal transporters (Log rank; *p* = 0.0003 and 0.005, respectively). A Cox proportional hazards model showed independent association of PET category classification with cardiovascular event.

**Conclusions:**

Peritoneal permeability expressed as PET category at peritoneal dialysis initiation is an independent cardiovascular risk factor in peritoneal dialysis patients using biocompatible peritoneal dialysis solution with neutral pH. Greater peritoneal permeability at peritoneal dialysis initiation might reflect subclinical vascular disorders.

**Electronic supplementary material:**

The online version of this article (doi:10.1186/1471-2369-15-173) contains supplementary material, which is available to authorized users.

## Background

Cardiovascular disease (CVD) is the most common cause of death in end-stage renal disease (ESRD) patients. Cardiovascular mortality in ESRD patients is about 10–20 times higher than among the general population [1]. It is noteworthy that CVD causes not only high mortality but also disabilities, which make it difficult for patients to continue peritoneal dialysis (PD) therapy by themselves. Physical disability caused by CVD affects the selection of dialysis modality, which is one of the possible reasons for the lack of increase of PD patients [2]. Therefore, the management of CVD and its risk factors is a primary goal for PD patients [3]. Several non-traditional cardiovascular risk factors specific for PD therapy are known, such as decreased residual renal function, ultrafiltration failure, and peritoneal protein loss [3–5].

Although these findings were derived mainly from data of patients using conventional PD solutions (CPDSs) with low pH, biocompatible PD solutions (BPDSs) with neutral pH have been widely used currently. Nevertheless, risk factors of CVD in PD patients using BPDSs remain unclear. This study investigated a patient cohort to clarify the association of clinical parameters at PD initiation, especially those specific for PD patients including peritoneal transport status, with cardiovascular event occurrence in patients using BPDS.

## Methods

### Patients and data collection

This retrospective cohort study used data of PD patients obtained from medical records at a single center: The University of Tokyo Hospital. The Institutional Review Board of The University of Tokyo approved the study. Because this study retrospectively collected the data available from the medical records, the Institutional Review Board of The University of Tokyo waives the requirement to obtain documentation of the consent.

In actuality, 137 adult patients started PD therapy during 2001–2009. After excluding patients who had received maintenance hemodialysis or kidney transplantation before PD initiation and those who had been followed up for less than 6 months after PD initiation, 102 patients were analyzed in this study. All patients underwent PD using glucose-based BPDS with neutral pH and devices provided by Terumo Corp. (Tokyo, Japan). Within 6 months after PD initiation, peritoneal membrane function by peritoneal equilibration test (PET), urinary and peritoneal solute clearance, and peritoneal protein excretion were evaluated in all patients. In PET, 2.5% glucose concentration 2-L volume PD solution was used. The ratio of creatinine concentration in dialysate to plasma at the completion of the 4 h dwell period (D/Pcre) was evaluated to estimate low-molecular-weight solute transport [6]. All the patients were classified into PET categories by the value of D/Pcre [6]. Then they were divided into three groups: low (L) transporters (D/Pcre <0.50), low-average (LA) transporters (0.50 ≤ D/Pcre <0.65), and high-average to high (HA + H) transporters (D/Pcre ≥0.65). The body weight and blood pressure were measured on the same day of PET. Patient data at PD initiation were collected, including age, gender, cause of ESRD, and CVD history before PD initiation.

Laboratory data were measured at the time of PET. Residual renal function and dialysis dosage were calculated as the weekly Kt/V from the 24 h urinary and dialysate clearance by direct measurement of urea in urine and dialysate. To evaluate peritoneal protein excretion, peritoneal protein clearance (P-PrCl) was calculated as described previously [5, 7]. Occurrence of cardiovascular event (CV event) after PD initiation was examined based on medical records and was evaluated as a primary outcome. CV event was defined for this study as follows: coronary artery disease which needs to be treated by angioplasty or coronary artery bypass, cerebrovascular disease such as transient ischemic attack and stroke, and peripheral artery disease that requires surgical revascularization or amputation, which occurred within five years after PD initiation. Technique survival within five years after PD initiation and death from PD initiation to the first 1 year after PD cessation were also examined.

### Statistical analysis

Continuous and categorical data were expressed respectively as means ± SD and proportions. Data were compared using Mann–Whitney u-test, chi-square test, and Cochran-Armitage test. Incidence of CV event, mortality, and technique survival rate were examined by comparing patients divided into PET categories using the Kaplan–Meier method and the log-rank test. Predictors of CV event were identified using the Cox proportional hazards model with variables that were significantly different between groups with and without CV event with a *p* value of less than or equal to 0.01. A *p* value of less than 0.05 was inferred as statistically significant.

Statistical analyses were conducted using software (JMP 9.0; SAS Institute Inc., Cary, NC).

## Results

### Patient data and characteristics

Table 1 presents patient data and characteristics at PD initiation. All 102 patients were treated with glucose-based PD solutions with neutral pH (MIDPERIQ L® 135 or MIDPERIQ L® 250; Terumo Corp.). For this study, patients were classified into groups: 19 (19%) into the L group, 59 (58%) into the LA group, and 24 (24%) into the HA + H group (HA group, n = 18; H group, n = 6) (Table 1). Of the 102 patients, 91 (89%) were treated continuously by automated PD (APD) from immediately after PD initiation to cessation of PD. In these patients, APD instead of continuous ambulatory PD (CAPD) was selected according to patient preferences. Laboratory data collection was performed more than 1 month after PD catheter insertion to avoid the influence of surgery. The recommended period to perform first PET after PD initiation is within 3 months [8]. In this study, the mean (± standard deviation) duration from PD initiation to laboratory data collection was 2.9 ± 1.6 months.

**Table 1:** **Patient characteristics at PD initiation (** ***N =*** **102, mean ± SD)**

Age (years)	60.6 ± 13.9	Serum albumin (g/dl)	3.5 ± 0.5
Male gender (*n* [%])	78 [77%]	Blood urea nitrogen (mg/dl)	52.1 ± 14.7
Cause of ESRD (*n* [%])		Serum creatinine (mg/dl)	6.6 ± 2.5
Diabetes	35 [34%]	Serum corrected calcium (mg/dl)	8.9 ± 1.1
Glomerulonephritis	34 [33%]	Serum phosphate (mg/dl)	4.6 ± 1.1
Nephrosclerosis	15 [15%]	C-reactive protein (mg/dl)	0.43 ± 0.68
Others	18 [18%]	Total cholesterol (mg/dl)	198 ± 39
Diabetes mellitus (*n* [%])	35 [34%]	Triglyceride (mg/dl)	173 ± 129
CVD before PD initiation (*n* [%])	25 [25%]	Renal weekly Kt/V	1.14 ± 0.62
Automated PD (*n* [%])	91 [89%]	Total weekly Kt/V	2.23 ± 0.58
Body mass index (kg/m^2^)	22.1 ± 3.4	D/Pcre	0.59 ± 0.12
Systolic blood pressure (mmHg)	131 ± 18	PET category	
Diastolic blood pressure (mmHg)	77 ± 12	L	19 [19%]
Urine volume (ml/day)	1182 ± 612	LA	59 [58%]
UF volume (ml/day)	446 ± 658	HA + H	24 [24%]
Hemoglobin (g/dl)	10.7 ± 1.6	P-PrCl (ml/day)	58.9 ± 26.9

ESRD, end stage renal disease; CVD, cardiovascular disease; UF, ultrafiltration; D/Pcre, the ratio of creatinine concentration in dialysate to plasma at the completion of the 4 h dwell period in peritoneal equilibration test; PET, peritoneal equilibration test; P-PrCl, peritoneal protein clearance.

### Occurrence of CV event and death after PD initiation

A CV event occurred in 18 patients (17.6%) within five years after PD initiation. Table 2 presents variables for which a significant difference was found between groups with and without a CV event. Age, prevalence of CVD before PD initiation, C-reactive protein, D/Pcre, and P-PrCl were significantly higher, and hemoglobin and serum albumin were significantly lower in the group of patients with CV event than in the group of patients without CV event (Table 2). Distributions of patients classified into each PET category differed significantly between groups with and without CV event (Table 2).

**Table 2:** **Comparison between patient groups with or without cardiovascular event within five years after PD initiation**

Variables	Cardiovascular event (mean ± SD)		***p***value
	(−) ***N =*** 84	(+) ***N =*** 18	
Age (years)	59.1 ± 14.3	67.4 ± 9.7	< 0.01
Male gender	75%	83%	0.45
Cause of ESRD			0.67
Diabetes	31%	50%	
Glomerulonephritis	36%	22%	
Nephrosclerosis	15%	11%	
Others	18%	17%	
Diabetes mellitus	31%	50%	0.13
CVD before PD initiation	20%	44%	0.03
Automated PD	88%	94%	0.43
Body mass index (kg/m^2^)	22.1 ± 3.4	21.7 ± 3.3	0.73
Systolic blood pressure (mmHg)	130 ± 18	140 ± 18	0.07
Diastolic blood pressure (mmHg)	77 ± 12	75 ± 12	0.24
Urine volume (ml/day)	1166 ± 579	1258 ± 763	0.57
UF volume (ml/day)	477 ± 640	286 ± 745	0.31
Hemoglobin (g/dl)	10.8 ± 1.7	10.2 ± 1.1	0.03
Serum albumin (g/dl)	3.6 ± 0.5	3.3 ± 0.5	0.01
Blood urea nitrogen (mg/dl)	52.5 ± 14.1	50.1 ± 17.2	0.49
Serum creatinine (mg/dl)	7.0 ± 2.6	5.7 ± 2.3	0.08
Serum corrected calcium (mg/dl)	8.9 ± 1.2	9.0 ± 0.7	0.90
Serum phosphate (mg/dl)	4.7 ± 1.1	4.3 ± 1.3	0.10
C-reactive protein (mg/dl)	0.39 ± 0.65	0.63 ± 0.83	0.05
Total cholesterol (mg/dl)	198 ± 40	201 ± 38	0.87
Triglyceride (mg/dl)	175 ± 138	164 ± 74	0.49
Renal weekly Kt/V	1.16 ± 0.65	1.12 ± 0.52	0.99
Total weekly Kt/V	2.23 ± 0.60	2.19 ± 0.50	0.88
D/Pcre	0.58 ± 0.12	0.65 ± 0.11	< 0.01
PET category			< 0.01
L	23%	0%	
LA	60%	50%	
HA + H	18%	50%	
P-PrCl (ml/day)	54.7 ± 24.2	72.7 ± 34.1	0.03

Of 102 patients, 15 patients (15%) were confirmed to have died between PD initiation and 1 year after PD cessation. Distributions of patients classified into each PET category differed significantly between the groups with and without death (Table 3).

**Table 3:** **Comparison between patient groups with or without death after PD initiation**

Variables	Death (mean ± SD)		***p***value
	(−) ***N =*** 87	(+) ***N =*** 15	
Age (years)	58.0 ± 12.8	75.3 ± 11.0	< 0.01
Male gender	78%	67%	0.33
Cause of ESRD			0.05
Diabetes	33%	40%	
Glomerulonephritis	39%	0%	
Nephrosclerosis	11%	33%	
Others	16%	27%	
Diabetes mellitus	34%	40%	0.62
CVD before PD initiation	24%	27%	0.85
Automated PD	91%	80%	0.22
Body mass index (kg/m^2^)	22.2 ± 3.3	21.3 ± 3.8	0.36
Systolic blood pressure (mmHg)	130 ± 17	134 ± 24	0.48
Diastolic blood pressure (mmHg)	78 ± 11	67 ± 14	< 0.01
Urine volume (ml/day)	1222 ± 619	948 ± 530	0.11
UF volume (ml/day)	484 ± 649	249 ± 693	0.21
Hemoglobin (g/dl)	10.8 ± 1.7	10.2 ± 1.2	0.04
Serum albumin (g/dl)	3.6 ± 0.4	3.1 ± 0.5	< 0.01
Blood urea nitrogen (mg/dl)	53.0 ± 14.4	46.6 ± 15.4	0.11
Serum creatinine (mg/dl)	6.9 ± 2.5	4.5 ± 1.9	< 0.01
Serum corrected calcium (mg/dl)	8.9 ± 1.2	9.1 ± 0.7	0.55
Serum phosphate (mg/dl)	4.8 ± 1.1	3.9 ± 0.8	< 0.01
C-reactive protein (mg/dl)	0.40 ± 0.69	0.61 ± 0.60	< 0.01
Total cholesterol (mg/dl)	199 ± 39	193 ± 41	0.58
Triglyceride (mg/dl)	178 ± 136	147 ± 73	0.86
Renal weekly Kt/V	1.13 ± 0.63	1.23 ± 0.58	0.57
Total weekly Kt/V	2.21 ± 0.56	2.30 ± 0.73	0.61
D/Pcre	0.58 ± 0.11	0.67 ± 0.15	0.03
PET category			< 0.01
L	21%	7%	
LA	61%	40%	
HA + H	18%	53%	
P-PrCl (ml/day)	56.3 ± 27.9	67.5 ± 17.3	0.02

### Impact of PET category classification at PD initiation on the prognosis of PD patients

**Figure 1:**
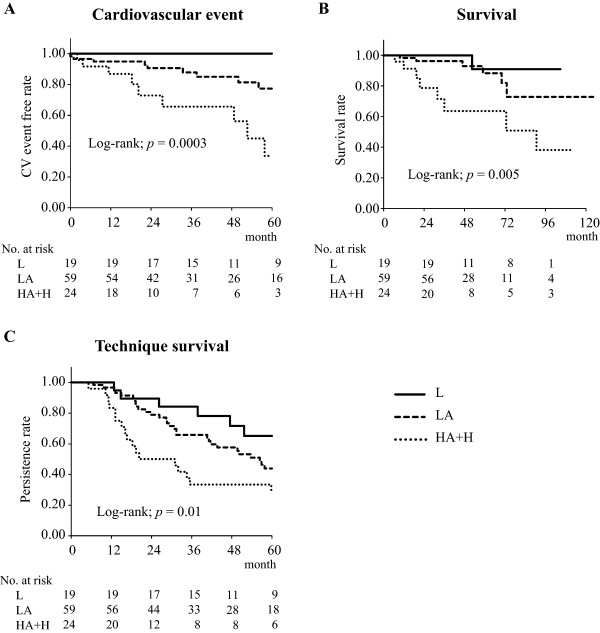
**Kaplan–Meier plots of incidence of CV event (A), survival rate (B), and technique survival rate (C) of PD patients using BPDS with neutral pH according to PET category.** 102 patients were divided into three groups based on PET category defined by D/Pcre. Patients with D/Pcre <0.50 are in the low (L) group (*n =* 19), 0.50 ≤ D/Pcre <0.65 are in the low average (LA) group (*n =* 59), and D/Pcre ≥0.65 are in the high average and high (HA + H) group (*n =* 24).

The incidence of CV event, survival rate, and technique survival rate were examined by comparing patients in the L, LA, and HA + H groups using the Kaplan–Meier method and the log-rank test (Figure 1). Patients in the HA + H group had worse CVD morbidity, survival rate, and technique survival rate than those in the L or LA group. Patients in the HA + H group had significantly lower levels of serum albumin and higher P-PrCl than those in the L or LA group (Table 4).

**Table 4:** **Comparison between groups divided according to PET category defined by D/Pcre**

Variables	PET category (mean ± SD)			***p***value
	L	LA	HA + H	
	***N***= 19	***N =*** 59	***N =*** 24	
Age (years)	60.4 ± 15.5	58.9 ± 13.7	64.9 ± 13.0	0.24
Male gender	68%	78%	79%	0.66
Cause of ESRD				0.74
Diabetes	26%	32%	46%	
Glomerulonephritis	52%	32%	21%	
Nephrosclerosis	11%	15%	17%	
Others	11%	20%	17%	
Diabetes mellitus	26%	32%	46%	0.54
CVD before PD initiation	16%	24%	33%	0.62
Automated PD	90%	88%	92%	0.99
Body mass index (kg/m^2^)	20.9 ± 2.8	22.6 ± 3.5	21.8 ± 3.5	0.18
Systolic blood pressure (mmHg)	126 ± 14	132 ± 18	134 ± 22	0.31
Diastolic blood pressure (mmHg)	75 ± 12	78 ± 11	75 ± 15	0.54
Urine volume (ml/day)	1155 ± 401	1260 ± 684	1012 ± 543	0.49
UF volume (ml/day)	654 ± 440	437 ± 616	300 ± 868	0.24
Hemoglobin (g/dl)	11.2 ± 1.2	10.6 ± 1.9	10.6 ± 1.2	0.28
Serum albumin (g/dl)	3.8 ± 0.4	3.6 ± 0.4	3.3 ± 0.5^*,**^	< 0.01
Blood urea nitrogen (mg/dl)	50.8 ± 15.8	54.2 ± 13.8	47.9 ± 15.3	0.26
Serum creatinine (mg/dl)	6.5 ± 2.0	6.9 ± 2.6	5.9 ± 2.7	0.28
Serum corrected calcium (mg/dl)	9.1 ± 0.8	8.8 ± 1.3	9.0 ± 0.7	0.66
Serum phosphate (mg/dl)	4.7 ± 1.4	4.9 ± 1.0	4.1 ± 1.0^**^	< 0.01
C-reactive protein (mg/dl)	0.38 ± 0.83	0.41 ± 0.62	0.54 ± 0.73	0.09
Total cholesterol (mg/dl)	197 ± 27	195 ± 44	207 ± 37	0.35
Triglyceride (mg/dl)	227 ± 215	161 ± 105	160 ± 69	0.39
Renal weekly Kt/V	1.26 ± 0.56	1.14 ± 0.60	1.09 ± 0.74	0.58
Total weekly Kt/V	2.21 ± 0.53	2.20 ± 0.54	2.32 ± 0.73	0.95
D/Pcre	0.45 ± 0.05	0.57 ± 0.05^*^	0.75 ± 0.10^*,**^	< 0.01
P-PrCl (ml/day)	44.2 ± 23.4	56.0 ± 24.9	74.1 ± 27.8^*,**^	< 0.01

*p <0.05 vs. L group; **p <0.05 vs. LA group.

Results of the Cox proportional hazards model on CV event in five years after PD initiation are shown (Table 5). Regarding forward stepwise multivariate analysis, the PET category remained as an independent predictor of CV event (Table 5). When analysis using the Cox proportional hazards model was conducted using variables that were significantly different between the patient groups with and without CV event with *p* value of less than or equal to 0.05, the PET category was determined as an independent predictor of CV event (Table 5).

**Table 5:** **Univariate and multivariate Cox regression model on cardiovascular event within five years after PD initiation**

		Unadjusted		Adjusted	
	Variable	Hazard ratio [95% CI]	***p***value	Hazard ratio [95% CI]	***p***value
Model 1	Age	1.03 [0.99–1.06]	0.09	1.03 [0.99–1.06]	0.09
	PET category	3.70 [1.62–8.43]	< 0.01	3.70 [1.62–8.43]	< 0.01
Model 2	Age	1.01 [0.98-.1.05]	0.40	–	–
	Serum albumin	0.29 [0.08-1.02]	0.05	0.24 [0.08-0.77]	0.02
	PET category	3.21 [1.37-7.52]	< 0.01	3.49 [1.49-8.15]	< 0.01
Model 3	Age	1.03 [0.99–1.08]	0.13	1.03 [0.99–1.07]	0.11
	CVD before PD initiation	1.82 [0.58–5.69]	0.31	–	–
	Hemoglobin	0.91 [0.66–1.26]	0.57	–	–
	Serum albumin	0.40 [0.09–1.83]	0.24	0.31 [0.09–1.11]	0.07
	C-reactive protein	1.19 [0.55–2.61]	0.65	–	–
	PET category	4.65 [1.78–12.16]	< 0.01	4.25 [1.79–10.10]	< 0.01
	P-PrCl	0.99 [0.97–1.01]	0.54	–	–

## Discussion

This study demonstrated that peritoneal small solute permeability, expressed as PET category classification defined by D/Pcre, was independently associated with a CV event within five years after PD initiation in patients using BPDS. This report is the first describing evaluation of the relation between clinical parameters at PD initiation and prognosis in patients using BPDS with neutral pH, although many previous clinical studies evaluated this relation in patients using CPDSs and icodextrin with low pH.

Our results showed that PET category at PD initiation is an independent risk factor of CVD in PD patients using BPDF with neutral pH. Similar relations between peritoneal permeability and prognosis shown by our study have been demonstrated in previous studies in patients using CPDSs with low pH [9–11]. Because PD solutions have adverse effects on both the local peritoneal and systemic effects induced by glucose degradation products coupled with other factors such as low pH, BPDSs had been anticipated for use to improve the prognosis of PD patients [12]. However, a recent systematic review was unable to demonstrate significant effects of BPDSs on survival and technique failure [13]. Results obtained from the present study support the view that the type of PD solution does not have a significant effect on the PD patient prognosis. Although the effects of BPDSs on prognosis remain unclear, some benefits have been reported. For instance, BPDSs have benefits of reducing peritoneal membrane injury, which might be favorable for the preservation of peritoneal ultrafiltration capacity and which might decrease the risk of CVD caused by fluid overload [14]. Reportedly, BPDSs can preserve residual renal function and levels of markers of endothelial dysfunction [13, 15–17]. Because local and systemic effects of BPDSs are preferred to those of CPDSs, the fluid overload state, which plays a pivotal role in CVD progression, is expected to be improved by BPDSs [3, 12]. The impact of BPDSs and clinical factors affecting the prognosis of patients using BPDSs should be investigated further.

Some possible explanations about the linkage between peritoneal permeability and prognosis of PD patients have been considered. High peritoneal permeability, which causes rapid equilibration of glucose across the peritoneal membrane, engenders ultrafiltration failure. A continuous fluid overload state accelerates left ventricular hypertrophy and increases serum concentrations of natriuretic peptides, which are markers of CVD and all-cause mortality in PD patients [3]. High peritoneal transport increases CVD morbidity and mortality by accelerating hypoalbuminemia, a well-known prognostic marker in PD patients, through increased peritoneal protein loss [9, 10]. Increased peritoneal permeability is probably associated with high mortality in patients with malnutrition–inflammation–atherosclerosis (MIA) syndrome through peritoneal protein loss and hypoalbuminemia [18]. High transport status of the peritoneum might be a marker of preexisting vascular pathology associated with atherosclerotic comorbidity after PD initiation [19].

Several studies have revealed that high peritoneal permeability is not an independent risk factor of death and technique failure. In addition, high transporter treated by APD had superior survival and technique survival [20–22]. Actually, APD tends to be selected for patients with increased peritoneal permeability because short exchange of PD solution by automated cycler can increase ultrafiltration via reduction of glucose absorption [22]. In our study, almost all patients were treated by APD from immediately after PD initiation to cessation of PD whether their peritoneal permeability was increased or not. Therefore, the PET category at PD initiation might predict the prognoses of patients treated by APD.

Although classification into ‘Slow’ (D/Pcre <0.55–0.60), ‘Average’, and ‘Fast’ (D/Pcre >0.80) transporter status was recently recommended for prescription management in clinical practice [23], classification into the four PET categories (L, LA, HA, and H) has been used in many studies, as it was for this study. We divided the patients into the three categories of L, LA, and HA + H in this study. However one previous study, in which the HA group comprised most of the patients, showed no difference of CV event rate among the PET category groups of L + LA, HA, and H [22]. When the patients in this study were divided into L + LA, HA, and H groups, or Slow, Average, and Fast groups, the Kaplan–Meier method and the log-rank test showed that the patients in the L + LA group or the Slow group of this study had a significantly high CV event free rate, patient survival rate, and technique survival rate (Additional file 1: Figures S1 and S2). Therefore, the association of peritoneal transport status with prognosis of PD patients using BPDS was apparently similar, irrespective of the patient categorization based on the PET results.

Our study has some limitations. This study was a retrospective cohort study of patients from a single center and the number of patients was not large. A large prospective study including patients using BPDS from multiple centers should be conducted. Because only glucose-based BPDS was used and because APD was mainly performed in this study, it remains unclear whether all the results from our study are applicable to patients using other PD solutions or treated by CAPD.

## Conclusions

Peritoneal permeability expressed as PET category defined by D/Pcre at PD initiation is an independent cardiovascular risk factor in patients using BPDS. Increased peritoneal permeability at PD initiation might contribute to the progress of atherosclerosis in PD patients by several mechanisms such as fluid overload and MIA syndrome. Findings from this study can contribute to management of PD patients using BPDS by enabling identification of patients with high risk of CVD and by enabling early intervention to improve their prognosis from immediately after PD initiation.

## Electronic supplementary material

Additional file 1: **Kaplan–Meier plots of incidence of CV event** **(Figures S1A and S2A)** **, survival rate** **(Figures S1B and S2B)** **, and technique survival rate** **(Figures S1C and S2C)** **of PD patients according to PET category.** 102 patients were divided into three groups based on PET category defined by D/Pcre. Patients with D/Pcre <0.65 are in the low and low-average (L + LA) group (*n =* 78), 0.65 ≤ D/Pcre ≤0.80 are in the high-average (HA) group (*n =* 18), and D/Pcre >0.80 are in the high (H) group (*n =* 6) **(Figure S1).** Patients with D/Pcre <0.57 are in the Slow group (*n =* 48), 0.57 ≤ D/Pcre ≤0.80 are in the Average group (*n =* 48), and D/Pcre >0.80 are in the Fast group (*n =* 6) **(Figure S2).** (PDF 190 KB)
