# Supplementary figures and images for: Increased peritoneal permeability at peritoneal dialysis initiation is a potential cardiovascular risk in patients using biocompatible peritoneal dialysis solution
Source: BMC Nephrol. 2014 Nov 1;15:173. doi: 10.1186/1471-2369-15-173 (PMC4230909; doi:10.1186/1471-2369-15-173)

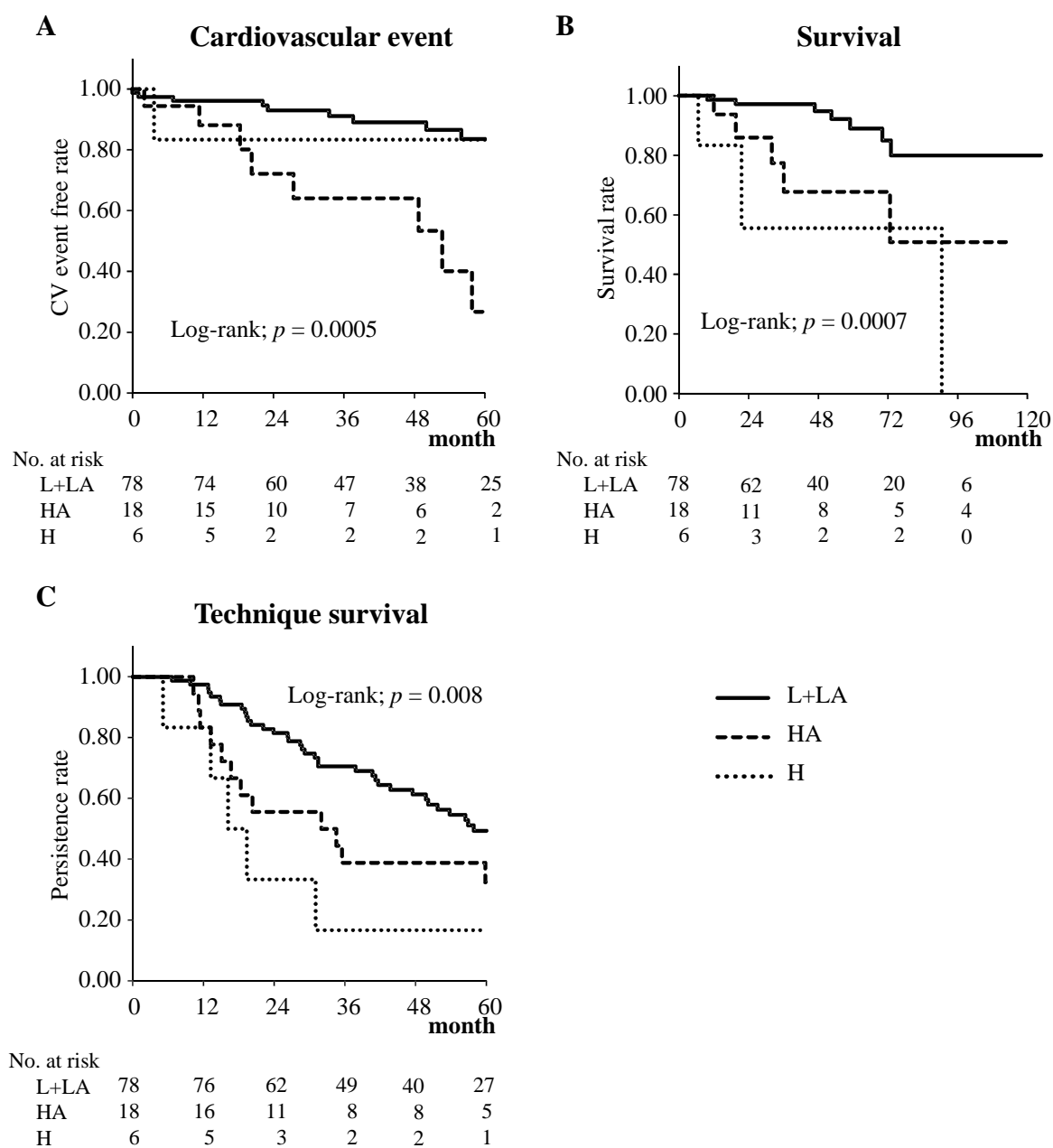

Figure S1

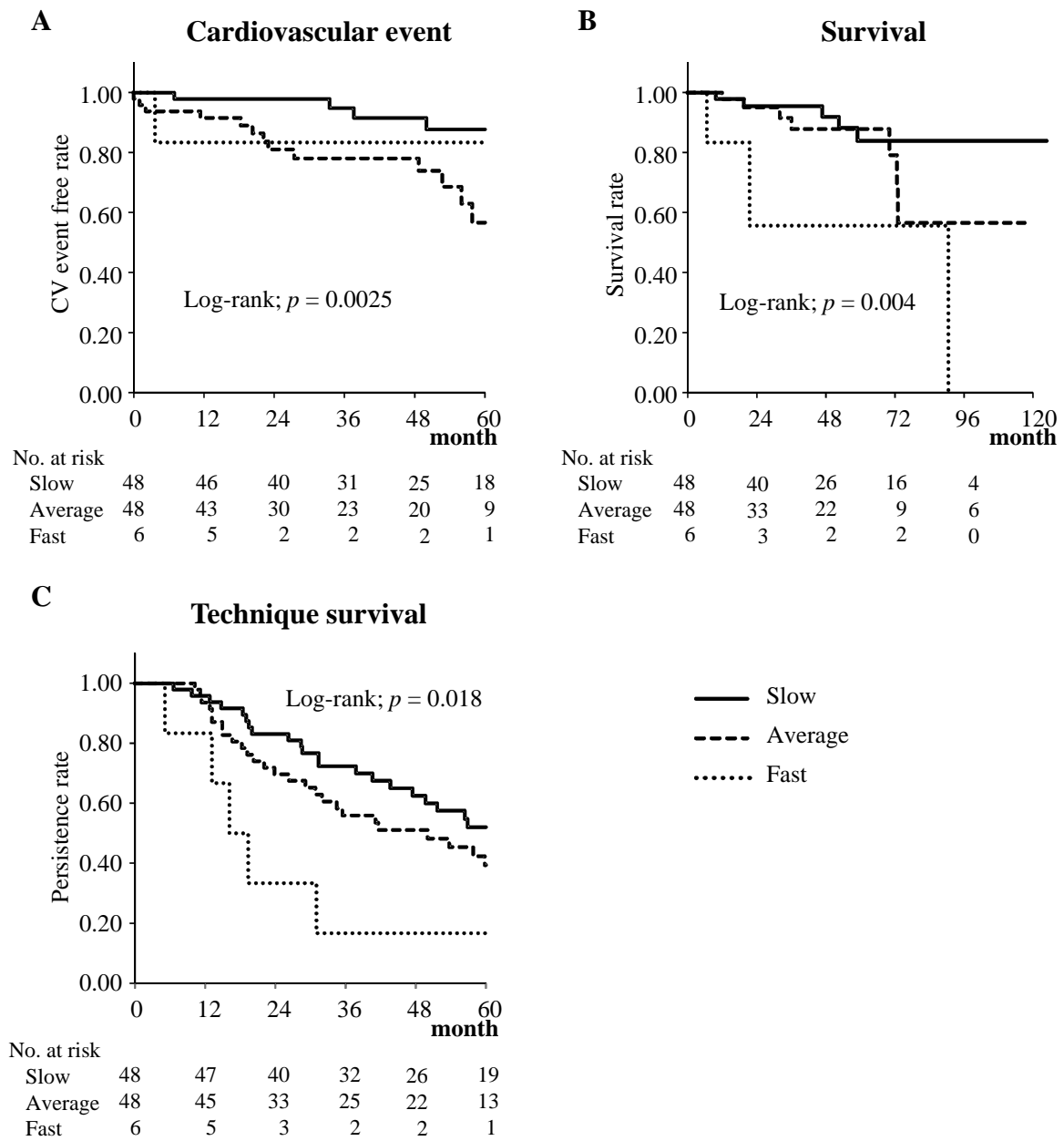

Figure S2

Supplement: Supplementary file 1 — Additional file 1: Kaplan–Meier plots of incidence of CV event (Figures S1A and S2A) , survival rate (Figures S1B and S2B) , and technique survival rate (Figures S1C and S2C) of PD patients according to PET category. 102 patients were divided into three groups based on PET category defined by D/Pcre. Patients with D/Pcre <0.65 are in the low and low-average (L + LA) group (n = 78), 0.65 ≤ D/Pcre ≤0.80 are in the high-average (HA) group (n = 18), and D/Pcre >0.80 are in the high (H) group (n = 6) (Figure S1). Patients with D/Pcre <0.57 are in the Slow group (n = 48), 0.57 ≤ D/Pcre ≤0.80 are in the Average group (n = 48), and D/Pcre >0.80 are in the Fast group (n = 6) (Figure S2). (PDF 190 KB) [file 12882_2014_862_MOESM1_ESM.pdf]
